# Giant hepatic hemangioma in a patient with cirrhosis: challenging to manage

**DOI:** 10.4322/acr.2024.485

**Published:** 2024-04-04

**Authors:** Marlone Cunha-Silva, Clauber Teles Veiga, Larissa Bastos Eloy da Costa, Simone Reges Perales, Amanda Avesani Cavotto Furlan, Elaine Cristina de Ataíde, Ilka de Fátima Santana Ferreira Boin, Tiago Sevá-Pereira

**Affiliations:** 1 Universidade Estadual de Campinas (Unicamp), Divisão de Gastroenterologia (Gastrocentro), Campinas, SP, Brasil; 2 Universidade Estadual de Campinas (Unicamp), Departamento de Patologia, Campinas, SP, Brasil; 3 Universidade Estadual de Campinas (Unicamp), Departamento de Cirurgia, Campinas, SP, Brasil

**Keywords:** Case Reports, Hemangioma, Liver Cirrhosis

## Abstract

Giant hepatic hemangiomas are occasional in patients with cirrhosis. It remains a challenge to decide on the need for treatment and choose the most appropriate intervention. A 62-year-old woman was recently diagnosed with cirrhosis and complained of upper abdominal fullness, reduction in oral food intake, and weight loss of 6 kg over the last three years. Upper digestive endoscopy evidenced thin-caliber esophageal varices and significant extrinsic compression of the lesser gastric curvature. Abdominal computed tomography revealed an exophytic tumor in the left hepatic lobe, measuring 11.5 cm, which had progressive centripetal contrast enhancement from the arterial phase, compatible with hepatic hemangioma. Serum tumor markers were negative, and her liver function was unimpaired. The patient underwent surgical resection (non-anatomical hepatectomy of segments II and III) which had no immediate complications, and the histopathological evaluation confirmed cavernous hepatic hemangioma. Two weeks later, she was admitted to the emergency room with jaundice, signs of hepatic encephalopathy, and moderate ascites, and was further diagnosed with secondary bacterial peritonitis. As no perforations, abscesses, or fistulas were observed on subsequent imaging tests, clinical management was successfully carried out. This case highlights that giant hepatic hemangiomas may be symptomatic and warrant treatment. In the setting of cirrhosis and portal hypertension, physicians should be aware of the risk of hepatic decompensation following surgical resection, even in patients with Child-Pugh class A.

## SHORT COMMUNICATION

Hemangiomas are the most common benign tumors of the liver, with an incidence of 0.4% to 20% in autopsies.^1-3^ Women are more often affected,^4^ and small nodules are diagnosed incidentally by abdominal ultrasound (US), as they are frequently asymptomatic. Most hemangiomas measure less than 2 or 3 cm and are located mainly in the right hepatic lobe, barely detected by Doppler evaluation.^2,5^ The prevalence of hepatic hemangiomas in patients with cirrhosis is lower than in the general population (1.2% to 1.7%). Giant hemangiomas are occasional in this group.^3,5^

Giant hepatic hemangioma (HH) is considered when over 5 or 10 cm.^2-4^ It may lead to increased abdominal volume, pain, or discomfort and progress to severe complications such as bleeding or Kasabach–Merritt syndrome.^4,6,7^ Therapeutic options are challenging, especially in patients with advanced liver fibrosis.

A 62-year-old woman complained of upper abdominal fullness, reduction in oral food intake, and weight loss of 6 kg over the last three years. She was referred to our tertiary center after undergoing an abdominal US that showed cirrhosis, splenomegaly, and a heterogeneous liver tumor measuring 10 cm. She was diagnosed with metabolic syndrome and had no history of drinking alcohol. Her body mass index was 24.3 kg/m^2^ and she was on atenolol, losartan, indapamide, metformin, sulpiride, gliclazide, omeprazole, and alprazolam.

On physical examination, no signs of chronic liver disease were found, but there was a palpable and painless mass up to 5 cm below the xiphoid appendix. Viral serologies and autoantibodies were negative, and serum tumor markers were normal. Laboratory tests of liver and kidney function were also unimpaired, as shown in Table 1.

**Table 1:** Initial laboratory tests and liver function scores

**Laboratory test**	**Result**	*Normal range*	**Immunological test**	**Result**
Hemoglobin	9.6	12-16 g/dL	HBsAg	negative
Leucocytes count	4.57	4 - 10 x10^3^ /μL	Anti-HBc	negative
Platelets count	45	150 - 400 x10^3^ /μL	Anti-HBs	negative
ALT/AST	17 / 18	< 35 U/L / < 35 U/L	Anti-HCV	negative
AP/GGT	50 / 97	< 104 U/L / < 40 U/L	Anti-HAV IgG	positive
Total bilirubin	1.38	< 1.2 mg/dL	Anti-HAV IgM	negative
INR	1.08	< 1.25	ANA	negative
Creatinine	0.87	< 1.02 mg/dL	Anti-HIV	negative
Sodium	141	135-145 mEq/L	LIVER FUNCTION	
Albumin	4.0	3.5-5.2 g/dL	Child-Pugh class	A5
AFP/CA19-9	2.16/14	< 7 ng/mL/< 37 U/mL	MELD-sodium score	9

AFP: alpha-fetoprotein; Ag: antigen; ALT alanine aminotransferase; ANA: antinuclear antibody; AP: alkaline phosphatase; AST: aspartate aminotransferase; CA19-9: carbohydrate antigen 19-9; GGT: gamma-glutamyl transferase; HAV: hepatitis A virus; HCV: hepatitis C virus; HBs/HBc: related to hepatitis B serology; HIV: human immunodeficiency virus; IgG: immunoglobulin G; IgM: immunoglobulin M; INR: international normalized ratio; MELD: Model for End-Stage Liver Disease.

**Figure 1:**
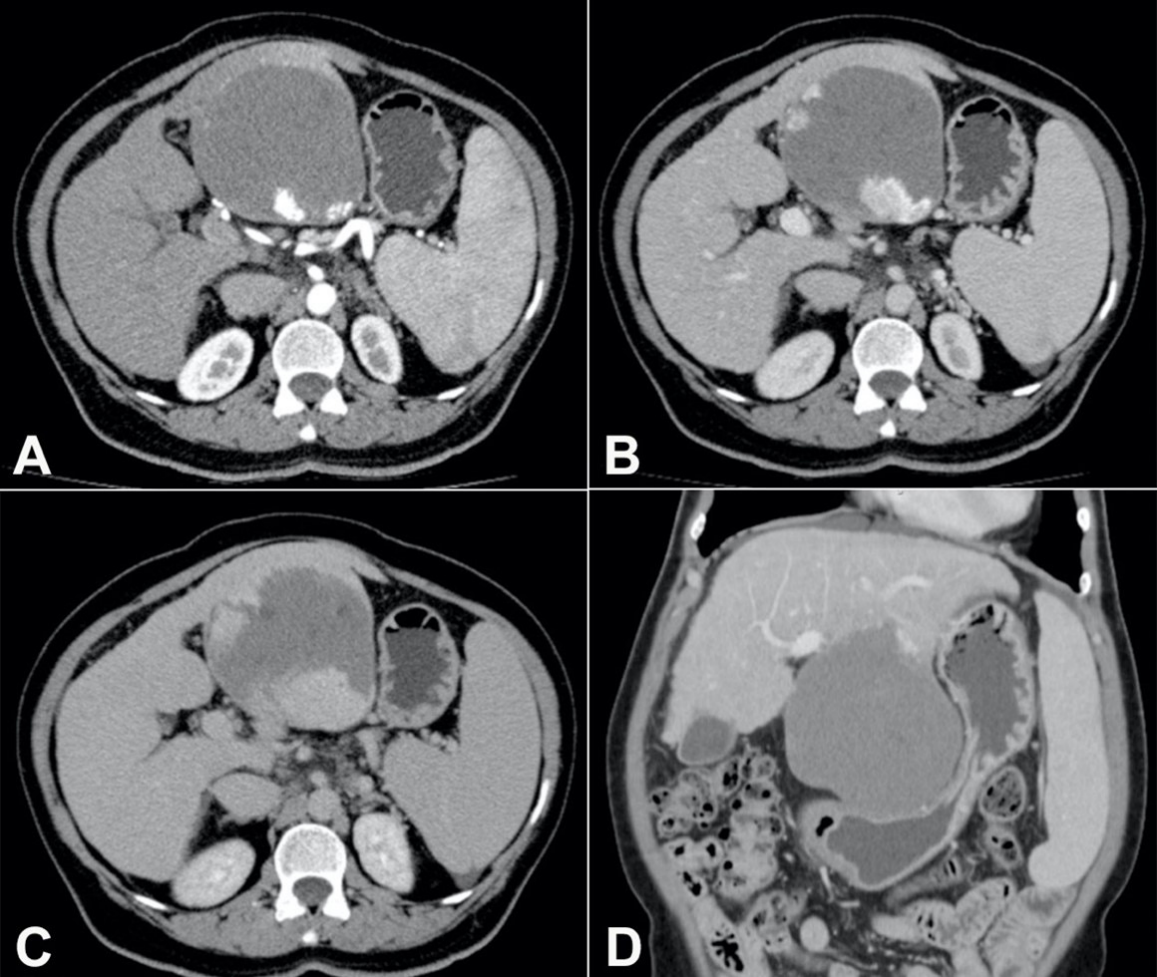
Contrast-enhanced abdominal computed tomography: axial section, in the (**A**) arterial, (**B**) venous, and (**C**) late phases: exophytic tumor in the left hepatic lobe (segments II and III), measuring up to 11.5 cm, with centripetal contrast enhancement, compatible with hepatic hemangioma; (**D**) coronal section, in the venous phase: hepatic hemangioma with significant compression of the stomach; liver with rounded borders and irregular surface, splenomegaly.

Upper digestive endoscopy evidenced two thin-caliber esophageal varices and significant extrinsic compression of the lesser gastric curvature. Computed tomography (CT) of the abdomen revealed an exophytic tumor in the left hepatic lobe measuring 11.5 cm in its largest diameter, with peripheral contrast enhancement in the arterial phase and progressive centripetal filling (Figures 1A to 1C), compatible with HH, which was compressing the stomach (Figure 1D) as observed on digestive endoscopy.

**Figure 2:**
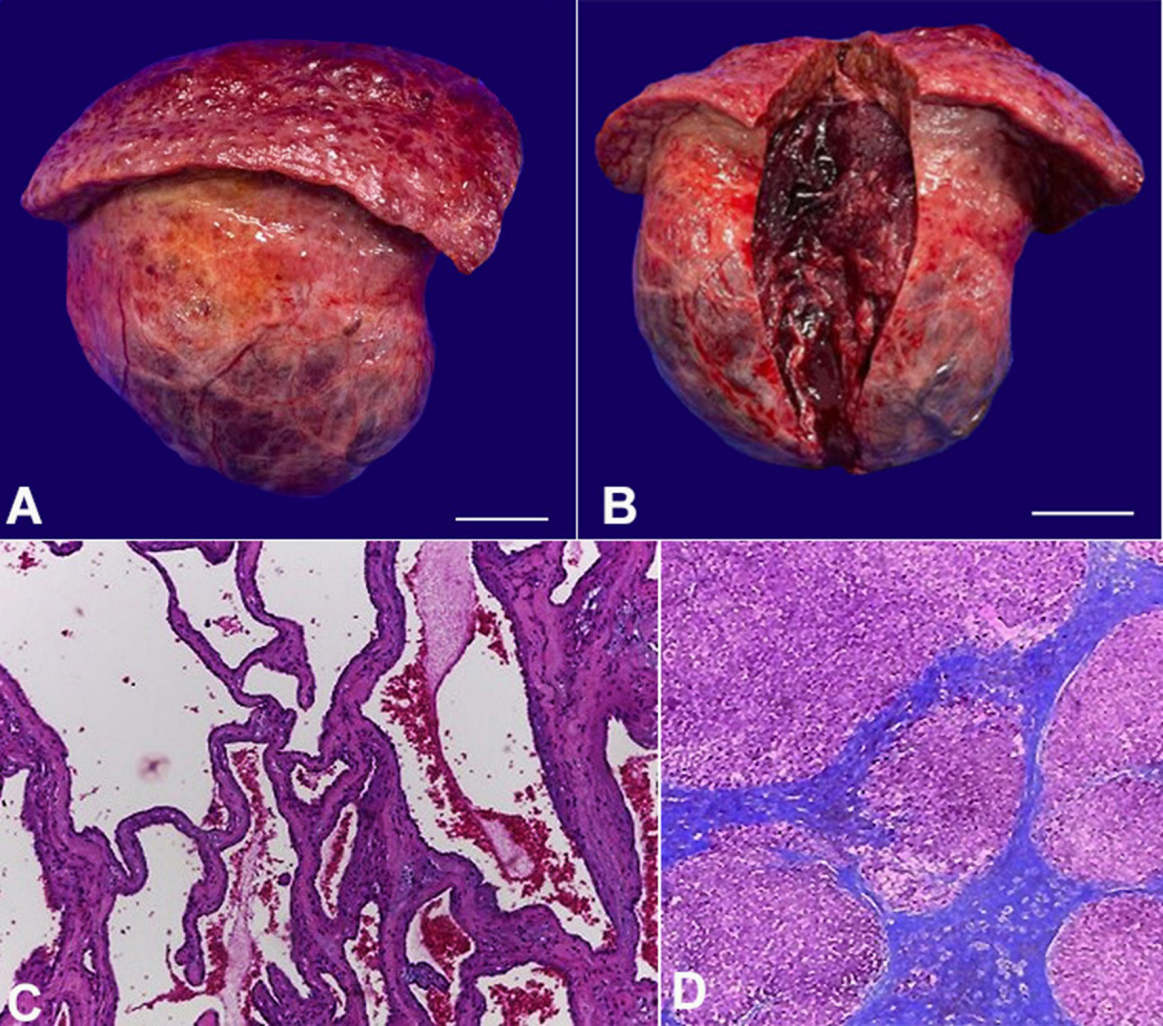
Product of hepatectomy: macroscopic evaluation: (**A**) external surface of the giant hemangioma and part of the left hepatic lobe, with a nodular surface (scale bar = 4 cm); (**B**) internal tissue of the tumor after sectioning (scale bar = 4 cm); microscopic analysis: (**C**) tumor: proliferations of blood-filled vascular channels of varying sizes, lined by single layers of flattened endothelial cells and separated by fibrous septa of different thickness, compatible with cavernous hepatic hemangioma [H&E, 20x]; (**D**) non-tumoral liver parenchyma: fibrotic septa that delimit regenerating nodules of hepatocytes, characterizing cirrhosis (Laennec stage 4C) [Masson's Trichrome stain, 4x].

The symptoms were attributed to the tumor. Considering the size and peripheral location, in the setting of cirrhosis and portal hypertension with thrombocytopenia, neither percutaneous radiofrequency ablation nor transarterial embolization was chosen. The patient underwent surgical resection (non-anatomical hepatectomy of segments II and III) (Figures 2A and 2B), which had no immediate complications. However, two weeks later, she was admitted to the emergency room with jaundice, signs of hepatic encephalopathy, and moderate ascites. Analysis of the ascitic fluid was compatible with secondary bacterial peritonitis, but no perforations, abscesses, or fistulas were observed on subsequent imaging exams. Clinical treatment was then performed, and the response was satisfactory.

Histological analysis of the resected mass revealed a cavernous HH (Figure 2C), and evaluation of the adjacent non-tumoral parenchyma showed cirrhosis (Laennec stage 4C) with macrovesicular steatosis and mild ballooning (Figure 2D). In the 2-year outpatient follow-up, she reported good acceptance of the oral diet with no complaints of upper abdominal discomfort. Gastric compression was no longer evident on the last abdominal CT scan, and her liver function was preserved (Child-Pugh class A6 and MELD-sodium score of 10).

Well-circumscribed and hyperechogenic nodules on the liver US are characteristic of HH, but contrast imaging is crucial in patients with cirrhosis to rule out hepatocellular carcinoma.^3-5^ On abdominal CT, HH shows peripheral contrast enhancement with gradual centripetal filling,^5^ as in our patient. Magnetic resonance imaging is superior to CT for diagnosing HH, which is usually hypointense/hyperintense on T1/T2 sequences, respectively. T1 sequences with gadolinium also show centripetal nodular filling from the arterial phase.^5^

Most hepatic hemangiomas are managed conservatively, including giant hemangiomas. A therapeutic approach must be considered in case of severe symptoms or complications.^6-8^ Percutaneous radiofrequency ablation and transarterial embolization are effective, but there is a lack of reports on the treatment of giant tumors in patients with cirrhosis.^6-9^ The main surgical procedures are enucleation, resection, and liver transplantation. Enucleation is safely performed for small and peripheral nodules while resection is chosen for lesions with an unclear border, as well as for a large tumor, especially when other hemangiomas surround it.^4,6^ It may also be indicated to rule out a malignancy when radiological exams are inconclusive.^1,7^ Liver transplantation might be considered in cases of multiple or giant hemangiomas requiring large-volume liver resection or in Kasabach–Merritt syndrome.^1,8^

To our knowledge, this is the largest HH ever reported in a patient with cirrhosis. Considering the stomach compression seen on CT scans and the improvement in abdominal discomfort after surgery, the HH was adjudicated as the source of her symptoms. Data on hepatic decompensation after surgical resection of HH in patients with advanced liver fibrosis are scarce. We could predict this risk by comparing it with resection of hepatocellular carcinoma, whose postoperative decompensation has already been associated with a MELD score higher than 9, extensive hepatectomies, and portal hypertension^10^ was most likely a determining factor in our case.

In conclusion, multiple aspects must be considered for the management of patients with cirrhosis and giant HH: I) the size, number, and location of the tumors; II) the association with severe symptoms or complications; III) liver function and the presence of portal hypertension; IV) the therapies available; and V) the medical team´s skills. In the scenario of portal hypertension, physicians should be aware of the risk of hepatic decompensation following surgical resection, even in patients with Child-Pugh class A.

## References

[1] Dong W, Qiu B, Xu H, He L (2019). Invasive management of symptomatic hepatic hemangioma. Eur J Gastroenterol Hepatol.

[2] Di Carlo I, Koshy R, Al Mudares S, Ardiri A, Bertino G, Toro A (2016). Giant cavernous liver hemangiomas: is it the time to change the size categories?. Hepatobiliary Pancreat Dis Int.

[3] Mastropasqua M, Kanematsu M, Leonardou P, Braga L, Woosley JT, Semelka RC (2004). Cavernous hemangiomas in patients with chronic liver disease: MR imaging findings. Magn Reson Imaging.

[4] Ribeiro MA, Papaiordanou F, Gonçalves JM, Chaib E (2010). Spontaneous rupture of hepatic hemangiomas: a review of the literature. World J Hepatol.

[5] Yu JS, Kim KW, Park MS, Yoon SW (2000). Hepatic cavernous hemangioma in cirrhotic liver: imaging findings. Korean J Radiol.

[6] Xie QS, Chen ZX, Zhao YJ, Gu H, Geng XP, Liu FB (2021). Outcomes of surgery for giant hepatic hemangioma. BMC Surg.

[7] Farhat W, Ammar H, Said MA (2021). Surgical management of giant hepatic hemangioma: a 10-year single center experience. Ann Med Surg.

[8] Eghlimi H, Arasteh P, Azade N (2020). Orthotopic liver transplantation for Management of a Giant Liver Hemangioma: a case report and review of literature. BMC Surg.

[9] Jiang T, Zhao Z, Cai Z, Shen C, Zhang B (2023). Case report: giant abdominal hemangioma originating from the liver. Front Oncol.

[10] Citterio D, Facciorusso A, Sposito C, Rota R, Bhoori S, Mazzaferro V (2016). Hierarchic interaction of factors associated with liver decompensation after resection for hepatocellular carcinoma. JAMA Surg.

